# Community Engagement, Ownership, and Civil Society Organizations in Polio Eradication

**DOI:** 10.4269/ajtmh.19-0529

**Published:** 2019-10

**Authors:** Jon K. Andrus, Henry B. Perry

**Affiliations:** 1 Division of Vaccines and Immunization, Center for Global Health, University of Colorado, Boulder, Colorado;; 2 Department of International Health, Johns Hopkins Bloomberg School of Public Health, Baltimore, Maryland

## INTRODUCTION

Since the 1988 resolution of the World Health Assembly of the World Health Organization to eradicate polio globally by 2000,^1^ the incidence of polio has declined more than 98%. Wild poliovirus type 2 has been eradicated and the eradication has been certified globally; more than 3 years have passed since the last wild poliovirus 3 was isolated in Nigeria. There are now three remaining reservoirs for wild poliovirus transmission—Nigeria, Afghanistan, and Pakistan.

This is no time to celebrate. The world is 19 years past the original target. The year 2019 in particular has not been kind to the Global Polio Eradication Initiative. During the first 6 months of 2019, the number of reported polio cases in Pakistan exceeded the number reported in 2018 over the same time period, and at present, the numbers are rising monthly.^2^ Parts of Afghanistan under Taliban control have stopped vaccination. Some communities in both Pakistan and Afghanistan actually refuse polio vaccination because of mistrust of the polio initiative, fueled by social media.^3^

Now, more than ever, community engagement and community ownership of the initiative, supported by civil society organizations in remaining areas at risk, must reverse the tide of public distrust and re-invigorate community ownership of efforts to fully vaccinate all children and ultimately reach the goal of the eradication of all polioviruses.^4^

Accordingly, the experience highlighted in the series of articles published in this supplement is incredibly timely. In the lead article, Losey et al. describe the model for community engagement for polio eradication supported by grants to the CORE Group (an association of U.S.-based non-governmental organizations (NGOs) working in community health) from the United States Agency for International Development (USAID) beginning in 1999 and later from the Bill & Melinda Gates Foundation. The CORE Group Polio Project (CGPP)^5^ worked with international and national NGOs to implement this program. Losey et al.^6^ state that such support has contributed to improvements in routine immunization programs, polio campaign quality, and surveillance for acute flaccid paralysis in many areas challenged by geography (being in isolated, dispersed, or remote areas), by the social characteristics of the population (being nomadic or transient), or by resistance to polio immunization (being resentful of the attention given to polio immunization while other basic health needs are being neglected, suspicious of the underlying motivation for immunization, or fearful of side effects falsely promoted for political purposes).^6^ Specifically, the authors describe how the CGPP developed innovative strategies to detect cases using community-based surveillance, promoted independent campaign monitoring, established cross-border initiatives, and developed a strong and creative cadre of Community Mobilizers (CMs) to track missed children, engage families in discussions about why immunizations are important, and link families to accessible vaccination sites.

Many strategies are required to reverse the tide of mistrust. Perhaps most important is listening to communities who doubt the benefit of polio eradication. As described by Solomon, this is exactly what was done in India.^7^ She relates how CGPP/India listened to the families and communities who refused to participate in the polio eradication program and then strategically addressed their concerns. This strategic approach, however, must also be well coordinated among the partners. Awale et al.^8^ discuss the various processes and techniques adopted in India to build strong partnerships and coordination mechanisms among stakeholders by optimizing their strengths and using opportunities that lead toward the eradication of polio. They go on to cite that such lessons in developing, managing, and nurturing partnerships can be adapted and replicated for elimination or controlling other diseases. Linked to this, data and mechanisms for sharing data are fundamental. Choudhary et al.^9^ describe how the community-based management information system (CB-MIS) has helped to make it possible for community-based health workers to understand barriers to immunization that families face and how to overcome them. The CB-MIS also provides a robust platform for community-based health workers to register vital events.

Any strategy is only as good as the quality of the staff at the point of implementation and the engagement of the community in the form of volunteers mobilized for support. Three articles from Ethiopia illustrate this point. Asegedew et al.^10^ describe the CGPP/Ethiopia, which began in 2001 to support polio eradication initiatives in hard-to-reach pastoralist and semi-pastoralist high-risk border areas of Ethiopia by training and supporting community volunteers (CVs) for immunization promotion and community-based surveillance activities. Their assessment of the program highlights the desire expressed by CVs to receive more training, suggesting a highly committed cadre of implementers. The authors note as a consequence the CVs have made notable contributions to polio eradication efforts in high-risk areas of Ethiopia, as well as to immunization promotion and disease control there. Using the trained network of volunteers, Tassema et al.^11^ also describe the Ethiopian experience. Their article suggests that the CGPP/Ethiopia was able to achieve increasing levels of polio immunization coverage in the hardest-to-reach areas of these states, with the achievement of levels actually higher than those achieved in the rest of Ethiopia. A third article, by Stamidis et al.^12^ report that CVs secured the buy-in of community members through open and fair eligibility and selection processes, which in turn ensured representation of community needs and perspectives. In this same article, the authors further describe how community-driven participation in Ethiopia consisted of identifying and choosing credible, trusted individuals who were willing to actively engage as caretakers of the community. Community volunteers then received specialized training and supportive supervision to build and expand their command of child health and vaccination information and interpersonal skills—fortifying the legitimacy of health messages and supporting the community’s sense of collective efficacy.

To that end, Nigeria also offers a rich experience. Usman et al.^13^ describe in their article how anti-immunization rumors are widely prevalent and serve as one of the greatest threats to the polio eradication program in North East Nigeria. The communication model of the CGPP, using community structures, has helped improve immunization acceptability in some of the most difficult areas in Nigeria’s states at the highest risk for polio. In another article from Nigeria, Duru et al.^14^ reveal that volunteer community mobilizers (VCMs) implemented the following innovative strategies to ensure high vaccination coverage: house-to-house mobilization; community dialogues; compound meetings; community health camps; and tracking of noncompliant families, missed children, and dropouts. The authors conclude that the involvement of VCMs in Nigeria’s polio initiative served as a pivotal contribution to reductions in the number of households rejecting polio immunization, the proportion of families with missed children, the proportion of families that were non-compliant, and the number of polio cases.

Ideally, all efforts should strive to impact health services and offer benefits well beyond polio. Chimpololo et al.^15^ write about how non-governmental health organizations in Malawi use a variety of approaches for social media, including mass media campaigns (radio and printed booklets), local skits and dramas, and home visits to secure not only success with polio eradication but also with the expansion of essential immunization coverage, the elimination of neonatal tetanus, and the control of measles. Every opportunity is taken to use training workshops and opinion leaders to impart knowledge and skills to CMs on immunization to eradicate polio, as well as to fight measles and neonatal tetanus. The authors conclude that major challenges faced by these non-governmental partners remain, including overcoming the demotivation of CMs because of lack of financial incentives.

Success to achieving disease targets is inextricably linked to innovative health capacity strengthening at all levels of the health system. An example is the article by Kisanga et al.^16^ describing the South Sudan experience of community-based surveillance, using their network of CVs. Such an approach complements the traditional facility-based surveillance system for acute flaccid paralysis, and it also provides opportunities for community engagement and ownership.

One guiding principle that has challenged the polio initiative over the decades is that progress must be sustained, inferring that all risks be mitigated. One such risk that continually haunts the program is the importation of wild poliovirus into previously polio-free countries, as has happened on repeated occasions in Africa, especially along border areas. To mitigate this risk, Arale et al.^17^ describe how project-trained CVs along the border of Kenya and Somalia have been a critical link between hard-to-reach communities and health facilities, as well as an excellent resource to support understaffed health facilities. The authors state that this Cross-Border Health Initiative (CBHI) has been effective in improving immunization coverage, disease surveillance, and rapid outbreak response in border areas. In addition, they conclude that the CBHI has the potential to address other public health threats that transcend borders.

Finally, in a summary article, Perry et al.^18^ describe how, despite numerous setbacks, the Global Polio Eradication Initiative has implemented community strategies with potential application for other global health issues. The authors argue that these strategies have the potential for contributing to the reduction of child and maternal mortality in hard-to-reach, underserved populations around the world. In addition, they emphasize how community-based surveillance, as developed by the CGPP, also has potential for improving global health security, now a global health priority.^19^ Global and regional policy and local action down to the household level, with all the necessary management and coordination steps in between (supervision, management, monitoring and evaluation, technical and operational cooperation, and research), will be required for these important benefits to be realized—benefits that will be well worth the effort and costs.

An important sub-theme of this series of articles is the organizational structure through which the CGPP has been implemented. USAID and the Gates Foundation provided funding to the Global Secretariat Office in Washington, DC (housed at World Vision/US), which provided funding to national secretariat offices, housed by a partner NGO, which then developed contracts with in-country NGOs to implement the program. As Kisanga rightly noted in his article in this series,^16^ “From its inception in 1999, the CGPP has developed a unique model of collaboration and coordination that transforms NGO contributions from small, isolated activities into large-scale collaborative interventions that optimize the intense, community-based programming of NGOs with the large-scale coordinated efforts needed to achieve polio eradication.” The country secretariats develop strategies for strengthening polio eradication activities in geographic areas defined as high risk by the in-country Interagency Coordinating Committee for Polio Eradication and then provide technical oversight, monitoring, and evaluation of activities of the CGPP’s NGO partners. This structure has great potential for replication in other initiatives to get evidence-based interventions down to households in high-need areas.

Most of the authors of the articles in this supplement are field program managers with limited experience in writing and publishing articles in peer-reviewed journals. It has been a great pleasure for us as co-editors and for our excellent anonymous peer reviewers to work with them in fashioning them into products that we hope you as readers learn from and enjoy the lessons described in this supplement, particularly as we work in the short term toward a world free of all polioviruses, and in the longer term toward a world free from deaths caused by readily preventable conditions.
